# Interstitial pneumonia and other adverse events in riluzole-administered amyotrophic lateral sclerosis patients: a retrospective observational study

**DOI:** 10.1186/s12883-019-1299-1

**Published:** 2019-04-27

**Authors:** Aya Inoue-Shibui, Masaaki Kato, Naoki Suzuki, Junpei Kobayashi, Yoshiki Takai, Rumiko Izumi, Yuuko Kawauchi, Hiroshi Kuroda, Hitoshi Warita, Masashi Aoki

**Affiliations:** Department of Neurology, Tohoku University Graduate School of Medicine Japan, 1-1 Seiryo-machi, Aoba-ku, Sendai, 980-8574 Japan

**Keywords:** Amyotrophic lateral sclerosis, Interstitial pneumonia, Riluzole, Liver dysfunction, Adverse events, Side-effect

## Abstract

**Background:**

Riluzole is the only approved oral drug for amyotrophic lateral sclerosis (ALS). We performed a retrospective study including ALS patients treated with riluzole, focusing on adverse events.

**Methods:**

Patients diagnosed with ALS according to the revised El Escorial criteria (World Federation of Neurology) in our center and who were administered 50 mg oral riluzole twice daily between January 2011 and September 2017 and followed up for at least 6 months from treatment initiation or until death were included. Data regarding sex, age, disease type, initial symptoms, biochemical analyses performed before and after riluzole administration, and medical history were collected. In case of withdrawal, cause of discontinuation and durations of disease and drug administration were recorded.

**Results:**

A total of 92 cases were enrolled. Riluzole administration was discontinued in 20 cases (21.7%). The most frequent reason for discontinuation was elevated liver enzymes (*n* = 5, 5.4%), followed interstitial pneumonia (IP), nausea and appetite loss, dizziness, general malaise, tongue paresthesia, and urinary urgency. In two cases, administration was discontinued primarily because of progression of bulbar palsy. All adverse events occurred within 6 months from treatment initiation and improved soon after its discontinuation. Three IP cases developed severe respiratory failure and required steroid treatment.

**Conclusion:**

Riluzole administration was discontinued in 20 cases among total of 92 cases. Careful follow-up is important for the first six months after the initiation of riluzole administration, including through interviews, chemical analyses, and chest X-rays, as required.

## Background

Amyotrophic lateral sclerosis (ALS) is a fatal neurodegenerative disease, primarily affecting upper and lower motor neurons. Glutamate excitotoxicity, which leads to neural damage, is one of the causative mechanisms [18]. Riluzole, the only approved oral drug for ALS, is a presynaptic glutamate release inhibitor, which may prevent neural damage and delay muscle-strength deterioration. Moreover, it decreases peak sodium (Na+) current and mediates voltage-gated Na + channel inactivation, which inhibit persistent Na + current in motor neurons, decreasing neuronal excitability and leading to neural protection [1, 18]. Although riluzole was well tolerated by and increased survival of ALS patients [4, 15], whether the survival benefit is elicited at the early or late stage or throughout the course of the disease remains controversial [7, 8, 14]. In terms of adverse events due to riluzole, few reports have focused on summative assessment [3, 4, 7, 8, 10, 15]. Effects of riluzole on the overall quality of life (QOL) of patients remain unknown. To investigate potential adverse events during riluzole treatment and to establish strategies to prevent or manage such events, we performed a retrospective study of ALS patients treated with riluzole, focusing on cases of withdrawal.

## Methods

The study was submitted to and approved by the Ethics Committee of Tohoku University Graduate School of Medicine (2010–253, 2017–1-005). Consent to participate was directly provided by patients or their families.

Patients diagnosed with ALS (clinically definite, clinically probable, clinically probable-laboratory supported, or clinically possible) according to the revised El Escorial criteria (World Federation of Neurology) in our center and who were administered 50 mg oral riluzole twice daily between January 2011 and September 2017 and followed up for at 6 six months from treatment initiation or until death were included. Data regarding sex, age, disease type, initial symptoms, biochemical analyses before and after riluzole administration, and medical events and histories were recorded. When alanine aminotransferase (ALT) and aspartate aminotransferase (AST) levels were > 3× the upper limit of the normal level [2, 12] and accompanied by additional subjective symptoms, such as anorexia, nausea, or dizziness, liver dysfunction was considered and riluzole was discontinued. In addition, in cases of withdrawal, causes of discontinuation and durations of disease and drug administration were recorded. Wald chi-square and t-test were used for data analysis. *P* < 0.05 was considered significant.

## Results

In total, 92 cases were followed up for a median of 15.5 months [interquartile range (IQR), 9–22 months]. Median patient age was 64.5 and 62.5 years in discontinuation and continuation cases, respectively (*P* = 0.35). The initial symptom was muscle weakness in almost all cases (discontinuation: 19/20; continuation 70/72), and cervical area was the most common site of symptoms (discontinuation: 9/20; discontinuation, 23/72; *P* = 0.5). Frequency of ALS/frontotemporal dementia was 20% (4/20) in the discontinuation cases (*P* = 0.77) and 12.5% (10/72) in the continuation cases. No patient characteristics were significantly associated with drug discontinuation (Table 1).

**Table 1:** Profiles of the ALS cases examined in this study

	Discontinued	Continued	P value
n	20	72	
Sex (male, n, %)	12, 60	34, 47.2	0.31
Age (Median, IQR, years old)	64.5 (58.3–72.8)	62.5 (56.5–69.3)	0.35
Initial symptom, n	Muscle weakness: 19	Muscle weakness: 70	
	Muscle cramp: 1	Muscle cramp: 1	
		FTD: 1	
Onset site of weakness (n, %)	B: 5, 25.0	B: 23, 31.9	0.4
	C: 9, 45.0	C: 23, 31.9	0.5
	T: 0, 0	T: 2, 2.8	0.5
	L:6, 30.0	L: 22, 30.6	0.63
FTD (n, %)	5, 20	10, 12.5	0.77

*B* bulbar, *C* cervical, *FTD* Frontotemporal dementia, *L* lumbar, *T*: thoracic, *P* values are obtained by the Wald chi-square test, based on the null hypothesis that the characteristic contributes to the discontinuation more than the other reasons in the discontinued group. *P* value of age is obtained by t-test, compared discontinued group with continued group

No patient characteristics were significantly associated with drug discontinuation

The most frequent cause of discontinuation was elevation of liver enzymes (*n* = 5/92, 5.4%) followed by interstitial pneumonia (IP), nausea or appetite loss, dizziness, general malaise, tongue paresthesia, or urinary urgency. In two cases, the drug was discontinued because of progression of bulbar palsy. Median disease duration was 2 years (IQR, 1–3; range, 0–20). All adverse events occurred within 6 months of riluzole initiation, with half of the events occurring within 14 days (Tables 2 and 3). Median duration of drug administration in continuation cases was 15.5 months (IQR, 9–22 months), with the longest duration of 4 years (Data not shown).

**Table 2:** Characteristics of the discontinued cases

No.	Sex	Onset	Dementia	Disease type	The reason of discontinuation of riluzole	Duration from the beginning of riluzole (days)	Duration of ALS at withdrawing riluzole (years)	Past histories
		Age, site						
1	M	60, L	–	ALS	Nausea	0–3	3	BPH, Depressive status, HT
2	M	77, C	–	ALS	IP	60	1	Past smoker, HT
3	F	75, B	–	ALS	Progression of bulbar palsy		3	Diabetes
4	F	64, C	+	ALS/FTD	Appetite loss	0–3	5	
5	M	77, C	+	ALS/FTD	Urinary urgency	0–3	2	OMI, Chronic gastritis
6	M	53, C	–	ALS	Progression of bulbar palsy		3	
7	M	63, B	–	ALS	Refusal		1	Ventricular aneurism, HT
8	M	75, L	–	ALS	Dizziness	90–120	20	Emphysema, BA, HT, CAVB
9	F	72, B	+	ALS/FTD	IP	150	1	HT, HL
10	F	67, L	–	ALS	General malaise	90	3	none
11	F	43, L	–	ALS	Chlamydia pneumonia s/o, IP n/r/o	14	4	none
12	M	53, L	–	ALS	Paresthesia of tongue	180	1	Psoriasis vulgaris
13	M	54, L	–	ALS	Elevated liver enzymes	3	2	Diabetes, HL
14	M	56, B	–	ALS	Elevated liver enzymes / dizziness	14	1	Reflux esophagitis, Heavy drinker
15	F	63, C	–	ALS	Dizziness	4	2	none
16	F	67, C	–	ALS	Elevated liver enzymes / General malais	30	5	HL
17	F	78, B	+	ALS/FTD	Appetite loss	9	1	HT, HL, Depressive status
18	M	68, C	–	ALS	Elevated liver enzymes	2–14	1	Diabetes, HT
19	M	65, C	–	ALS	Elevated liver enzymes / dizziness	0–14	1	Diabetes, HL
20	M	59, C	–	ALS	IP	60	2	Past smoker, HT

*BA* bronchial asthma, *BPH* Benign prostatic hyperplasia, *CAVB* complete arterial-ventricular block, *F*: female, *HT* Hypertension, *HL* Hyperlipidemia, *IP* interstitial pneumonia, *M* male, *N/R/O* not ruled out, *OMI* old myocardial infarction, *S/O* suspected of

+: having dementia, −: not having dementia

All cases with elevated liver enzymes that discontinued riluzole presented a history of medication for diabetes or hyperlipidemia

**Table 3:** Characteristics of the discontinued cases, categorized into events

The reason of discontinuation of riluzole	n (rate vs. all, %)	Sex (Male, %)	Onset of ALS	Duration from the beginning of riluzole (days, median, IQR)	Duration of ALS at withdrew riluzole (years, median, IQR)	Past histories (n)
			Age (years old, median, IQR)			
Elevated liver enzymes	5 (5.4)	80	65 (56–67)	11 (7.3–14)	1 (1–2)	Diabetes/HT/HL (4), Reflux esophagitis (1), Heavy drinker (1)
IP	4 (4.3)	50	65.5 (55–73.3)	60 (48.5–82.5)	2 (1.8–2.5)	past smoker (2)
Appetite loss / Nausea	3 (3.3)	33	64 (62–71)	2 (2–5.5)	3 (2–4)	Depressive status (2)
Dizziness	2 (2.2)	50	69 (66–72)	4 and 90–120, respectively	2 and 20 years, respectively	
General malaise	1 (1.1)	0	67	90	3	
Paresthesia of tongue	1 (1.1)	100	53	180	1	Psoriasis vulgaris
Urinary urgency	1 (1.1)	100	70	0–3	2	

The most frequent cause of discontinuation was elevation of liver enzymes (n = 5/92, 5.4%) followed by IP, nausea or appetite loss, dizziness, general malaise, tongue paresthesia, or urinary urgency. In two cases, the drug was discontinued because of progression of bulbar palsy. Median disease duration was 2 years (IQR, 1–3; range, 0–20). All adverse events occurred within 6 months of riluzole initiation, with half of the events occurring within 14 days

In this study, ALS patients who showed ALT and AST levels > 3× of the upper limit of the normal level [2, 12] and presented with accompanying subjective symptoms, such as anorexia, nausea, and dizziness, were considered cases/patients with abnormal elevated liver enzymes. In discontinuation cases, biochemical analyses revealed abnormally elevated liver enzymes in five cases and elevated Krebs von den Lungen-6 (KL-6) and surfactant protein (SP)-D levels in four pneumonia cases. All cases with abnormal elevated enzymes presented with a medical history of diabetes, hyperlipidemia, or hypertension and received medication for these conditions (Table 2). Furthermore, in two cases with elevated liver enzymes, biochemical analyses before riluzole administration showed moderately elevated γ-GTP levels (Tables 2 and 3). Two continuation cases showed slightly elevated liver enzyme levels. Overall, 8 of the 72 continuation cases showed mildly elevated liver enzyme levels (< 70 U/L) after drug administration, but there were no accompanying symptoms.

Adverse events improved soon after drug discontinuation, except in three cases of IP that required steroid treatment to achieve a good response. Two cases with elevated liver enzymes and severe IP are reported below.

### Case1

The patient complained of weakness in her left (Lt.) upper limb at the age of 68 years, which gradually spread to the other side. Moreover, she experienced cramps in her Lt. upper and lower limbs at the age of 70 years and was admitted to our hospital. She presented no relevant family history. She presented a medical history of hyperlipidemia and received medication for it. Neurological examination revealed moderate muscle atrophy and weakness in her Lt. upper and lower limbs and fasciculation in her upper limbs. Electromyography revealed fasciculation in the thoracic area. We diagnosed the patient with ALS and initiated treatment with 50 mg riluzole twice daily. After 2 weeks of riluzole initiation, a skin rash appeared and riluzole was withdrawn. Riluzole was resumed 3 weeks after day 1 of treatment. Then, she complained of general malaise. Biochemical analysis at day 30 of riluzole treatment revealed elevated AST from 21 to 50 U/L and elevated ALT from 25 to 88 U/L (Table 4). We suspected drug-induced hepatic injury and discontinued the medication. Her liver enzyme levels worsened at 1 month after drug discontinuation (AST, 65 U/L; ALT, 132 U/L) but gradually improved; her malaise disappeared within 2 months after riluzole discontinuation (Table 4).

**Table 4:** The course of biochemical analyses data of case1 with elevated liver enzymes

	AST	ALT	ALP	γ-GTP	LDH	T-Bil
Before riluzole treatment	21	25	189	15	187	0.8
When abnormality occurred	50	88	259	17	213	0.7
The worst biochemical data	65	132	238	37	229	0.8
After discontinuation	25	37	259	21	175	0.6

*ALP* alkaline phosphatase, *ALT* alanine aminotransferase, *AST* aspartate aminotransferase, *γ-GTP* gamma-glutamyl transpeptidase, *T-bil* total bilirubin

T-bil: mg/dL, Others: U/L

Biochemical analysis at day 30 of riluzole treatment revealed elevated AST from 21 to 50 U/L and elevated ALT from 25 to 88 U/L. The liver enzyme levels worsened at 1 month after drug discontinuation but gradually improved; the symptom disappeared within 2 months after riluzole discontinuation

### Case 2

**Fig. 1:**
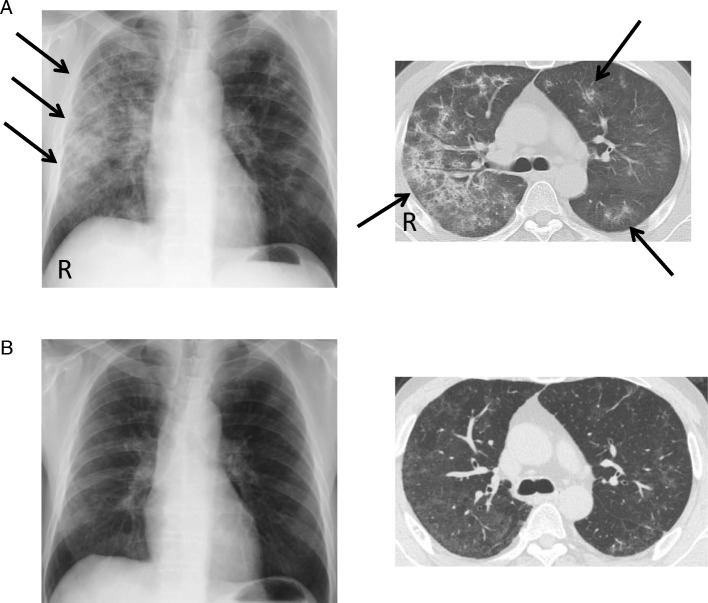
(**a**) Chest X-ray and CT radiography of the 61-year-old man of IP show consolidation in bilateral lungs, dominantly in the lower and right side (arrows). (**b**) On the 24th day after treatment with oral PSL, the consolidations are improved. R: right

The patient complained of fasciculation and muscle weakness in his upper limbs at the age of 59 years and was admitted to our hospital at the age of 60 years. He presented no relevant family history. He presented a medical history of proton pump inhibitor use. He was a past smoker of 30 cigarettes per day for 25 years. Neurological examination revealed hypertonus in his four extremities and muscle atrophy and weakness in his upper limbs. Electromyography revealed active denervation potentials in the cervical, thoracic, and lumbar areas. We diagnosed the patient with ALS and initiated treatment with 50 mg riluzole twice daily. The patient complained of shortness of breath and dry cough 2 months after treatment initiation. Physical examination revealed blood pressure of 105/75 mmHg and heart rate of 77 beats per minute. His SpO2 in room air was 92%. Routine biochemical analyses revealed increased KL-6 (1151 U/mL), SP-D (414 ng/mL), lactate dehydrogenase (354 U/L), C-reactive protein (0.9 mg/dL), and serum amyloid A (68.8 μg/mL) levels. Arterial blood gas analysis revealed hypoxemia with pO2 of 68.2 mmHg (Table 5). Chest X-ray and computed tomography (CT) revealed consolidation in the bilateral lower lung lobes (Fig. 1a). Pulmonary function test revealed diffusion impairment, with percent vital capacity (%VC) of 79.8%, forced expiratory volume percent in one second (FEV1.0%) of 70.4%, and diffusing capacity of the lung carbon monoxide (DLCO) of 49.2%. Drug-induced pneumonia was suspected, and riluzole treatment was withdrawn at day 80 of riluzole initiation. Bronchoalveolar lavage showed 57.8% increase in the lymphocyte counts. Transbronchial lung biopsy was performed from the right upper and lower segmental bronchi. Pathological analysis revealed organizing pneumonia—a subtype of IP. As the clinical course was acute and different from that of food microaspiration-induced idiopathic pulmonary fibrosis [19], we diagnosed the patient with drug-induced IP. and initiated oral prednisolone at 0.5 mg/kg body weight per day. Immediately, the symptoms and respiratory failure improved, with DLCO increasing to 105.3% and consolidation disappearing in 30 days (Table 5, Fig. 1b).

**Table 5:** The course of biochemical analyses and pulmonary function data of our IP case

Biochemical analysis		LDH	CRP	SAA	KL-6
	onset	354	0.9	68.8	1152
	after treatment	272	0.1	7.3	469
Blood gas analysis		pH	pCO_2_	pO_2_	HCO_3_
	onset	7.41	36.9	68.2	22.3
Pulmonary function test		% VC	FEV1.0%	DLCO	
	onset	79.8	70.4	49.2	
	after treatment	95.9	73.3	105.3	

*CRP* C-reactive protein, mg/dL, *DL*_*CO*_ Diffusing capacity of the lung carbon monoxide, ml/min/mmHg, *FEV*_*1.0%*_ Forced expiratory volume percent in one second, %, *KL-6* Krebs von den Lungen-6, *U/mL*, *LDH* lactate dehydrogenase, U/L, pCO_2_: mmHg, pO_2_: mmHg, *SAA* Serum amyloid A, *mg/mL, SP-D* surfactant proteins D, *ng/mL, %VC* Percent vital capacity, %,

On his admission, routine biochemical analyses revealed increased levels related to IP. Arterial blood gas analysis revealed hypoxemia. Pulmonary function test revealed diffusion impairment. The symptoms and respiratory failure improved, with DLCO increasing after discontinuation and steroid treatment

## Discussion and conclusion

Riluzole is a presynaptic glutamate release inhibitor, which protects motor neuron from toxic neural excitation. The most common adverse events due to riluzole is elevated liver enzyme levels, specifically ALT (6.9%), AST (6.6%), and γ-GTP (3.8%), as well as nausea (3.8%), according to a treatment outcome study in the pharmaceutical reference. In a previous study, ALT levels elevated 2–4 times the normal range in 7.8% of the cases and AST levels elevated 4 times the normal range in 14.2% of the cases were reported, with 6.5% of the cases showing elevation of both enzymes; 6.5% cases resulted in drug withdrawal in that study, and enzyme levels returned to normal within 2 months after riluzole discontinuation [4]. Another study reported elevated ALT in 6.7% cases and elevated AST in 3.8% cases [15]. In addition to liver enzyme levels, asthenia prevalence of 8.5% (versus 7.0% in placebo) and nausea prevalence of 4.9% (versus 3.5% in placebo) has been reported [3]. All adverse events, except for urinary urgency, noted in this study have been reported in previous research studies or pharmaceutical references (Table 3). All five cases with elevated liver enzyme (5.4%) showed elevated ALT and AST levels, whereas only two cases showed elevated γ-GTP levels; this trend is consistent with previously reported trends [4, 15]. A study investigating dose-dependent effects of riluzole has suggested that hepatotoxicity reflects metabolic toxicity of riluzole [15, 17]. In this study, all cases with elevated liver enzymes that discontinued riluzole presented a history of medication for diabetes or hyperlipidemia (Tables 2 and 3); this may reflect increased metabolic ability. In addition, in some cases, biochemical analyses before riluzole administration revealed elevated γ-GTP; this also suggests that metabolic ability before medication is important. Since biochemical analysis does not always indicate asthenia and symptoms sometimes resemble disease progression, repeated interviews to assess general fatigue would be useful for making decision to discontinue riluzole considering patient’s quality of life (QOL).

Although the incidence of riluzole-induced IP is 0.1% in Japan according to a pharmaceutical reference, the incidence rate in our study was much higher at 4.3%. Because ALS patients complain of dyspnea with disease progression, IP might be overlooked sometimes. The proposed mechanism underlying IP in riluzole-administered patients includes dosage-dependent cell-mediated allergy along with increased CD8-positive lymphocytes in bronchoalveolar lavage and DLST [5]. We speculate that IP in our cases was caused by riluzole-induced allergy. In case 2, prolonged history of smoking may have triggered IP. Therefore, conducting repeated interviews and chest X-rays, as required, are important to differentiate adverse events from disease progression.

A double-blind trial of riluzole has shown that AST levels increased after 42 to 267 days of treatment initiation [4]. A dose-ranging study of riluzole has reported that the median duration of AST increase was 51 days in the 100 mg-dosing group [15]. Some case reports have shown elevated liver enzymes after 3 weeks, 4 weeks, and 6 months of riluzole initiation [6, 17], while in some other case reports of IP, this duration was 3 weeks, 4 weeks, and 2 months [13, 20]. Based on a phase III clinical trial and drug-use survey performed for 18 months [15] and the abovementioned reports of adverse events occurring from 7 days to 9 months, we considered our follow-up period of a median of 15.5 months to be sufficiently long. Indeed, in our cases, all adverse events occurred within 6 months after riluzole initiation, suggesting that careful follow-up for the first 6 months after riluzole initiation is important. Despite a sufficiently long follow-up, this report was retrospective and duration of drug administration was variable. Further investigations including prospective data and cases with comparable starting points and follow-up periods are warranted. Moreover, a selection bias existed because of the single-center nature of this study, and studies including more patients from multiple centers are imperative.

All but three cases of IP, which required steroid treatment, showed improvement soon after drug discontinuation. Only one IP case not require steroid treatment was diagnosed as IP with ground glass opacity (GGO) on chest X-ray and represented a very early stage of the event. Although responses to steroids were good, the three IP cases could have been mild. Furthermore, the need for steroids could have been eliminated if patients had earlier detection, as in the GGO case.

Riluzole has been reported to be well tolerated for long periods of up to 7 years or more in the real-world setting [10, 14]. However, recurrent pancreatitis in two ALS patients associated with riluzole treatment has been reported recently [9]; both patients were diagnosed with pancreatitis within 3 months after riluzole initiation. Once again, these reports emphasize the importance of careful observation of adverse events in the first 6 months after riluzole administration.

Although survival benefits of riluzole are debatable, some reports have suggested early benefits such that riluzole induced partial normalization of cortical and peripheral axonal hyperexcitability in the early stage of ALS [11] or increased survival in the last clinical stage of ALS [10]. In the light of these reports, it is advisable to continue riluzole as long as possible unless QOL is affected by the adverse events. When QOL is affected due to adverse events, riluzole should be discontinued rather than tapering administration, which is supported by the fact that all but three cases of IP in this study showed improvement simply with riluzole discontinuation [7, 9, 14, 16].

IP could be treated and lethal in most cases. Therefore, all ALS patients should be carefully followed up through interviews after riluzole initiation, especially for the first 6 months. When patients complain of respiratory problems, such as dyspnea or dry cough, chest X-ray should be recommended.

Riluzole was discontinued in 20 ALS patients (20/92) in this study. Moreover, incidence of IP was higher in this study than that reported in past studies, and strategies to differentiate IP from disease progression are warranted. Finally, careful follow-up for the first 6 months after the initiation of riluzole treatment is crucial, including thorough interviews, chemical analyses, and chest X-ray, as required.

## Abbreviations

%VC: Percent vital capacity
ALS: Amyotrophic lateral sclerosis
ALT: Alanine aminotransferase
AST: Aspartate aminotransferase
CT: Computed tomography
DLCO: Diffusing capacity of the lung carbon monoxide
FEV1.0%: Forced expiratory volume percent in one second
FTD: Frontotemporal dementia
FVC: Forced vital capacity
GGO: Ground glass opacity
IP: Interstitial pneumonia
IQR: Interquartile range
KL-6: Krebs von den Lungen-6
LDH: Lactate dehydrogenase
Lt.: Left
N/R/O: Not ruled out
Na^+^: Sodium
PPI: Proton pump inhibitors
PSL: Prednisolone
QOL: Quality of life
S/O: Suspected of
SP: Surfactant proteins
